# Supplementary material for: Genomic Features and Evolution of the Parapoxvirus during the Past Two Decades
Source: Pathogens. 2020 Oct 27;9(11):888. doi: 10.3390/pathogens9110888 (PMC7694016; doi:10.3390/pathogens9110888)
Supplement: Supplementary file 1 [file pathogens-09-00888-s001.pdf]

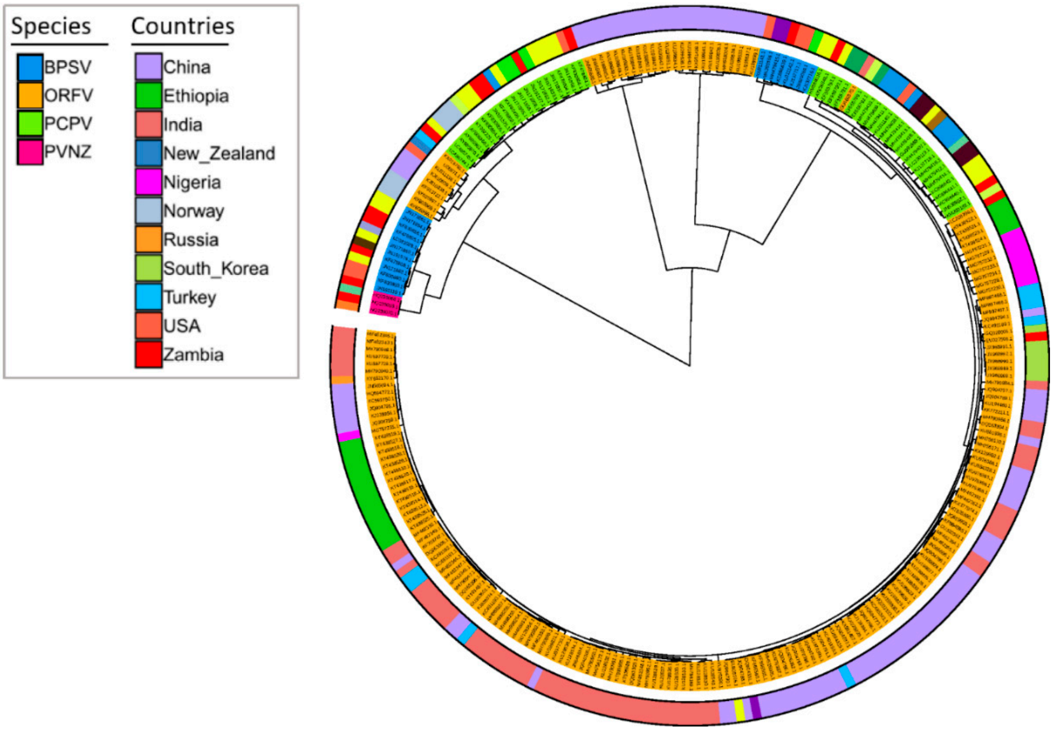

Figure S1. The phylogenetic tree of PPV B2L genes. The tree was generated by the maximum likelihood (ML) method using PAML v4.9. The tree was designed by using the online tool "iTOL". Here; different colors represent different collection locations. The color of each leaf node represents different virus species.
